# The Ability of Silicon Fertilisation to Alleviate Salinity Stress in Rice is Critically Dependent on Cultivar

**DOI:** 10.1186/s12284-022-00555-7

**Published:** 2022-02-02

**Authors:** Sarah J. Thorne, Petra M. Stirnberg, Susan E. Hartley, Frans J. M. Maathuis

**Affiliations:** 1 Department of Biology, University of York, York, YO10 5DD UK; 2 Department of Animal and Plant Sciences, University of Sheffield, Sheffield, S10 2TN UK

**Keywords:** Salt stress, Silicon, Cultivar, Rice, Cost–benefit analysis

## Abstract

Silicon (Si) fertiliser can improve rice (*Oryza sativa*) tolerance to salinity. The rate of Si uptake and its associated benefits are known to differ between plant genotypes, but, to date, little research has been done on how the benefits, and hence the economic feasibility, of Si fertilisation varies between cultivars. In this study, a range of rice cultivars was grown both hydroponically and in soil, at different levels of Si and NaCl, to determine cultivar variation in the response to Si. There was significant variation in the effect of Si, such that Si alleviated salt-induced growth inhibition in some cultivars, while others were unaffected, or even negatively impacted. Thus, when assessing the benefits of Si supplementation in alleviating salt stress, it is essential to collect cultivar-specific data, including yield, since changes in biomass were not always correlated with those seen for yield. Root Si content was found to be more important than shoot Si in protecting rice against salinity stress, with a root Si level of 0.5–0.9% determined as having maximum stress alleviation by Si. A cost–benefit analysis indicated that Si fertilisation is beneficial in mild stress, high-yield conditions but is not cost-effective in low-yield production systems.

**Supplementary Information:**

The online version contains supplementary material available at 10.1186/s12284-022-00555-7.

## Introduction

Silicon (Si) has long been recognised as a beneficial element for many plant species, especially members of the Poaceae (Epstein 1994; Debona et al. 2017; Luyckx et al. 2017). Members of the Poaceae accumulate relatively large amounts of Si; in rice (*Oryza sativa*) for example values as high as 10% Si by dry weight have been recorded (Epstein 1994). Much of this can be found in the form of silicon bodies, i.e. amorphous silica that is deposited in particular tissues, or in spines and other structures on the leaf surface (Hartley et al. 2015; Piperno 1988). Such high levels of accumulation and deposition suggest substantial benefits to plants from Si, but one consensus emerging from the literature is an absence, or only marginal effect of Si on plant growth in optimal, non-stress conditions (Cooke and Leishman 2016; Coskun et al. 2019). In contrast, in a number of species Si has been linked to increased resistance to pests and diseases (reviewed in Debona et al. 2017; Singh et al. 2020; Van Bockhaven et al. 2013) and also to improved tolerance to abiotic stress, notably drought and salinity (reviewed in Thorne et al. 2020).

Salt stress affects approximately 20% of all arable land (FAO and ITPS 2015). Ample Si supply, which can be achieved using Si fertilisation, can reduce salt stress in crops (Thorne et al. 2020). In rice, Si fertilisation is associated with increased anti-oxidative enzyme activity, which reduces the oxidative damage that occurs during salt stress (Das et al. 2018; Yan et al. 2020). Furthermore, Si can reduce the osmotic stress induced by salinity, which is correlated with changes in root morphology and osmotic potential (Yan et al. 2020). Salt induced depression of photosynthetic rates have also been shown to be partially reversed by Si (Farooq et al. 2015). Overall, these effects of Si are associated with improved growth and yield during salt stress (Ahmed et al. 2019).

The exact underlying mechanisms for the beneficial effects of Si during salinity stress are not clear but may be related to tissue specific Si deposition. In the roots, Si is mostly found in endo- and exo-dermal tissues where it could be integrated into the cell wall by cross linking with other wall components such as hemicelluloses, pectins, lignins and phenolics (Sakai and Thom 1979; Fleck et al. 2015; He et al. 2015). The ensuing physical barrier will limit both ion and water permeability, forcing a relatively large proportion to move via the symplast where flux control is far greater. Alternatively, Si could promote suberisation and lignification of the Casparian strip, for example by altering transcript levels of relevant genes (e.g. Hinrichs et al. 2017). Barrier formation and strengthening of the Casparian strip has been shown to block the apoplastic ‘bypass’ flow of ions such as Na^+^ (Yeo et al. 1999; Gong et al. 2006; Flam-Shepherd et al. 2018; Yan et al. 2021) and Cl^−^ (Shi et al. 2013) in the root, and could form a mechanistic explanation for the Si-induced reduction in the levels of harmful ions in the shoot.

These findings suggest that increased levels of Si fertilisation may provide a sustainable strategy to mitigate salinity-associated yield loss. However, the economic feasibility of such an approach is unclear and likely to critically depend on a large set of parameters (Singh et al. 2020; Thorne et al. 2020). Some of the most important ones would include the type and cost of Si fertiliser, quantitative data regarding the exact levels of Si that are required to maximise salt stress alleviation, the variety under cultivation, and the level of stress that is applied. We therefore studied a number of rice cultivars, including several that are widely cultivated, to analyse how their response to salinity, to Si supplementation, and the interaction between these factors, varied. Growing plants in both hydroponics and soil, critical values for root and shoot Si contents were determined and showed that cost–benefit ratios greatly vary according to growth conditions, rice cultivar, and production system. Costing models predict that Si fertilisation is beneficial in mild stress, high yield production systems but is not cost-effective in low yield production systems.

## Results and Discussion

### Different Cultivars Show Variation in Response to Salt Stress and Silicon

**Fig. 1:**
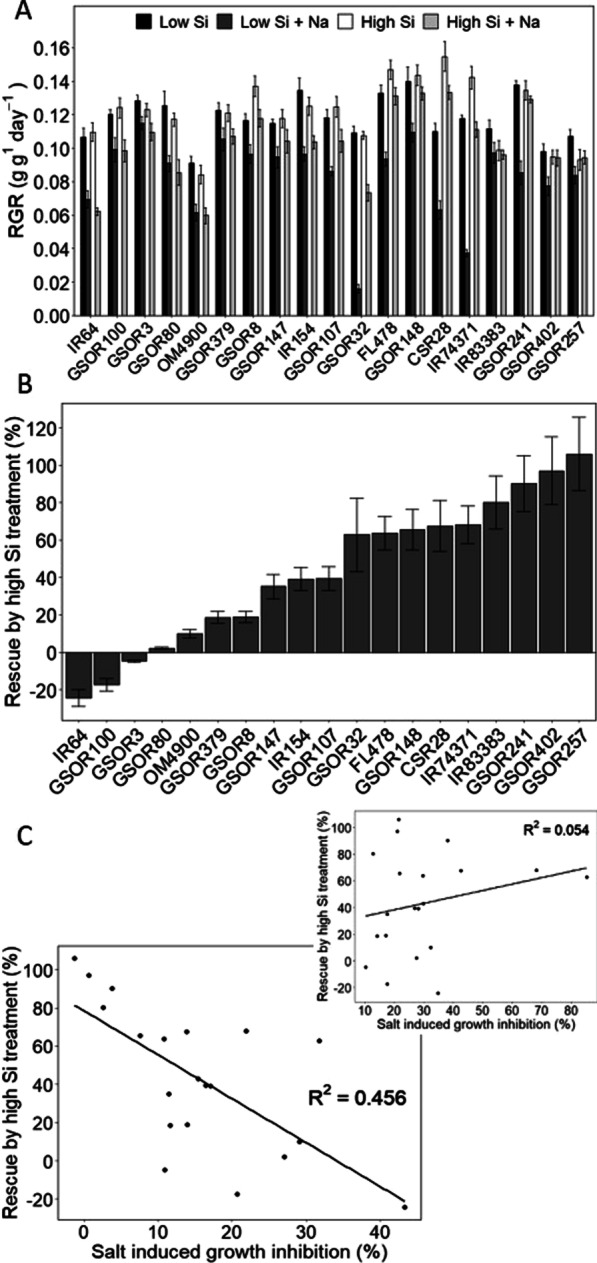
**a** Response of different rice cultivars to salinity and Si. Plants were grown in hydroponics for 30 d at low (0.07 mM) and high (1 mM) ambient Si, with (50 mM NaCl) or without (0 mM NaCl) salt stress. Data show means (N = 3–5) with standard deviations. **b** Increasing external Si concentration reduces the growth loss caused by salinity. When external Si was raised from 0.07 to 1 mM an overall reduction in growth loss was determined. However, the level of ‘rescue’ (salt-induced growth reduction at high Si relative to salt-induced growth reduction at low Si) varies greatly between rice varieties. **c** Si Rescue is greater in salt tolerant rice cultivars. Raising ambient levels of Si from 0.07 to 1 mM, lowers the salt-induced growth penalty but less so in salt-sensitive cultivars. Salt sensitivity-ranking was done at high external [Si]. Inset shows large variation in Si Rescue and lack of correlation with salt sensitivity when cultivars are ranked for sensitivity at low external Si condition

To test how the benefits of Si addition for alleviating salt stress varied between cultivars, nineteen cultivars were grown hydroponically at low (0.07 mM) and high (1 mM) ambient Si, without (0 mM NaCl) and with (50 mM NaCl) salt stress (Fig. 1a; cultivars listed in Additional file 1: Table S1).

A number of observations can be made on the outcomes of this experiment: first, there is little difference in the growth rate of cultivars under low vs high Si when salinity stress is absent, a phenomenon that has previously been reported (Yeo et al. 1999; Lekklar et al. 2019; Ahmed et al. 2019; Yan et al. 2021).

Second, raising external Si levels greatly improved plant resilience to salt stress, such that salinity-induced growth loses were approximately halved. Averaged across cultivars, growth rate reduction at low ambient Si was ~ 30%, whereas it was only ~ 15% at high ambient Si. Previous studies have focussed on overall biomass production rather than relative growth rates (RGR), but have nevertheless reported similar beneficial effects of Si (Flam-Shepherd et al. 2018; Lekklar et al. 2019; Ahmed et al. 2019).

Third, the salt sensitivity index (defined as salt-induced growth reduction with respect to control conditions) is not the same for the low and high Si treatments. For example, although the cultivar FL478 is traditionally defined as a salt tolerant variety (Walia et al. 2005), this is only evident at high external Si, whereas it shows considerable salt sensitivity at low Si. Likewise, Farooq et al. (2015) found that the growth of the selected salt-tolerant cultivar, KS-282, was more inhibited by salt treatment than the selected salt-sensitive cultivar, IRRI-6, when plants were grown without Si. Only when plants were supplemented with Si was the salt tolerance of KS-282 evident.

Fourth, a rise in ambient Si clearly benefits some cultivars more than others. Previous work that compared small numbers of cultivars with different salt tolerance is less clear: Farooq et al. (2015) found a stronger effect of Si on the salt tolerant cultivar than the sensitive cultivar, but Yeo et al. (1999) found that the effect of Si was more pronounced for a salt sensitive cultivar. We therefore compared a much larger number (nineteen) of cultivars. The percentage ‘rescue’ by Si (i.e. salt induced growth inhibition at high Si relative to that at low Si) varied from − 24% to 106% (Fig. 1b), demonstrating that Si actually exacerbated salt damage in some cultivars (for example in IR64). However, in others it (almost) restored growth to that observed in non-stress conditions, as can be seen for lines GSOR402 and 267. The amount of rescue strongly correlated with salt sensitivity in a negative manner (r =  − 0.68), but only in high Si conditions (Fig. 1c). When we used salt sensitivity values that were determined at low Si, a significant correlation was no longer observed (Fig. 1c inset). Thus, these results imply that the mitigating effect of Si on salinity stress is more pronounced in salt tolerant lines.

### Where Does Si Impact?

To assess whether the beneficial effects of Si depend on the levels deposited in roots, shoots or both, we analysed correlations between RGR and associated parameters on the one hand, and root or shoot tissue [Si] on the other. RGR was measured rather than yield as measuring yield requires soil-grown plants, from which it is not possible to obtain reliable root Si measurements. When grown in non-stress, control conditions, there was a consistent, substantial negative correlation between RGR and tissue Si. Although present when analysing root Si, this negative correlation was far stronger with shoot Si, and had r values of − 0.54 and − 0.69 respectively for low and high Si conditions (Additional file 1: Fig. S1a, b). When plants were grown in the presence of salt, even stronger (negative) correlations were observed between RGR and shoot Si with r values of − 0.73 and − 0.77 respectively for low and high Si conditions (Additional file 1: Fig. S1c, d). Thus, in rice at least, it appears that varieties that tend to accumulate Si in shoot tissue are relatively slow growing, and this phenomenon is insensitive to both ambient Si levels and (salinity) stress.

Theories of defence allocation on plants predict that Si accumulation should be greater in slower growing plant species and individuals (e.g. Massey et al. 2007). Such a trade-off between Si and growth was demonstrated across a range of crop species and their wild relatives: Simpson et al. (2017) found higher Si accumulation was associated with a lower growth rate, especially for larger plants. Other studies have likewise reported a negative correlation between Si accumulation and plant biomass (Johnson and Hartley 2018; de Tombeur et al. 2021). As yet, we have no mechanistic explanation for this observation; a substantial fraction of Si is translocated to the shoot by bulk flow through the xylem and perhaps transpiration fluxes are relatively large in slow growing plants. Alternatively, as Si accumulation involves the use of active efflux transporters (Ma et al. 2007; Ma and Yamaji 2015), there may be an energetic cost associated with high Si uptake (Simpson et al. 2017).

**Fig. 2:**
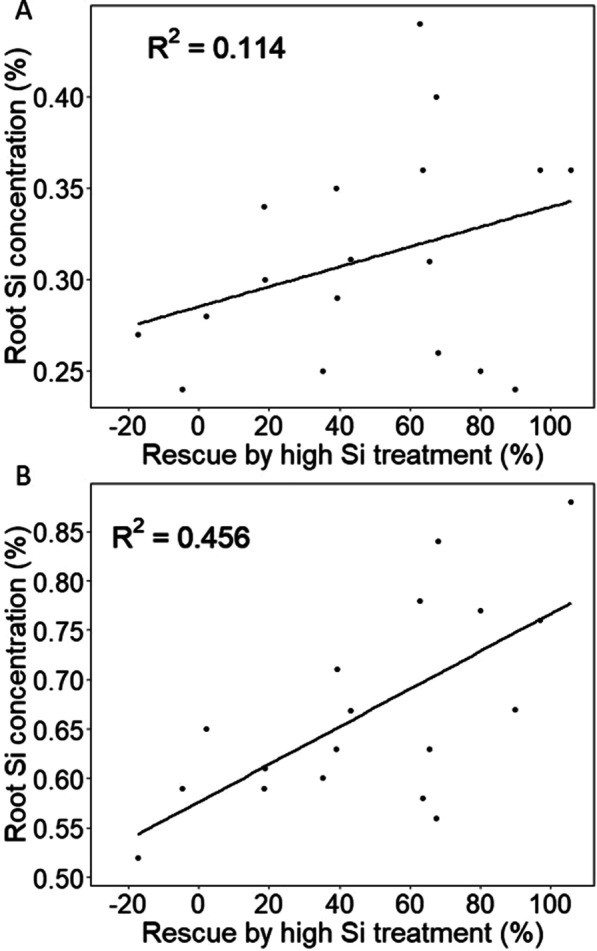
The mitigating effect of Si (% Rescue; defined as biomass in high Si plus salt condition relative to low Si plus salt condition) significantly correlates with root Si (**a**) but more so in high Si conditions (**b**)

Interestingly, the nature of these relationships drastically changed when the effect of Si on *salt sensitivity* was investigated, rather than on growth. Salt sensitivity, expressed as percent rescue (Fig. 1b), showed a significant positive correlation with root Si only (Fig. 2). Though this was rather weak for the low Si condition (r = 0.34), it substantially increased to an r value of 0.68 in plants grown in the high Si plus salt stress condition. These data strongly suggest that root Si rather than shoot Si is instrumental in improving salt tolerance in rice but the involved mechanism(s) are not clear. Si deposits around the root exo- and endodermis can strengthen the Casparian strip barrier function. This is particularly relevant in young roots and regions where lateral roots emerge because Casparian strips are not fully formed there, allowing considerable ‘leakage’ of Na^+^ and Cl^−^ ions via the apoplast (e.g. Gong et al. 2006; Flam-Shepherd et al. 2018). Average shoot Na^+^ levels were greatly reduced after Si supplementation (Table 1) from around 2000 to 1200 µmole g DW^−1^ which corroborates a model where Si reduces ionic bypass flow in the root and as such mitigates ionic toxicity stress in the shoot (Yeo et al. 1999; Gong et al. 2006; Flam-Shepherd et al. 2018; Yan et al. 2021). Such a scenario is supported by data that show no or very little effect of Si on shoot Na^+^ in OM4900 and IR64 (Additional file 1: Table S2), cultivars that do not respond to Si. Thus, in such cultivars bypass flow may be inherently low as was previously argued to be the case for the Si non-responsive Pokkali (Flam-Shepherd et al. 2018). However, we also identified cultivars with a large Si-induced reduction in shoot Na^+^, but nevertheless showed no, or a negative, response to Si supplementation (e.g. GSOR3 and GSOR108). In these cases, Si-independent salt sensitivity factors other than shoot Na^+^ may be more important such as maintaining gas exchange or adequate vacuolar sequestration of Na^+^ and Cl^−^ (e.g. Maathuis et al. 2014). Alternatively, the potential benefits of Si (e.g. lowered shoot Na^+^) may be negated by other Si effects such as increased transpiration causing water stress (Thorne et al. 2020) or adverse interaction with Na^+^ and Cl^−^ transporters (Flam-Shepherd et al. 2018).

**Table 1:** Tissue Na^+^ concentrations (µmole kg^−1^)

	Na (µmole kg^−1^)			
	Low Si		High Si	
	No Na	With Na	No Na	With Na
Shoot	145 ± 37	2019 ± 571	131 ± 43	1299 ± 535
Root	341 ± 48	768 ± 147	347 ± 52	712 ± 112

Plants were grown in hydroponics for 30 d at low (0.07 mM) and high (1 mM) ambient Si, with (50 mM NaCl) or without (0 mM NaCl) salt stress. Table show means with standard deviations. N = 3

### Critical Levels of Si for Maximum Stress Mitigation

The above data show that Si leads to an improvement of biomass production during salt stress, but the extent varies greatly between cultivars. To assess whether such variability is a function of cultivar-specific Si requirement, eight cultivars were tested on an expanded range of Si concentrations (0, 0.07, 0.4, 1 and 3 mM) and levels of salinity (0, 50 and 80 mM NaCl). This set included salt tolerant lines (GSOR3 and FL478), lines with medium tolerance (GSOR115, and the widely grown elites IR64 and OM4900), a well characterised drought tolerance trait donor (CSR28) and two salt sensitive, high yielding elite varieties (IR154 and IR74371).

**Fig. 3:**
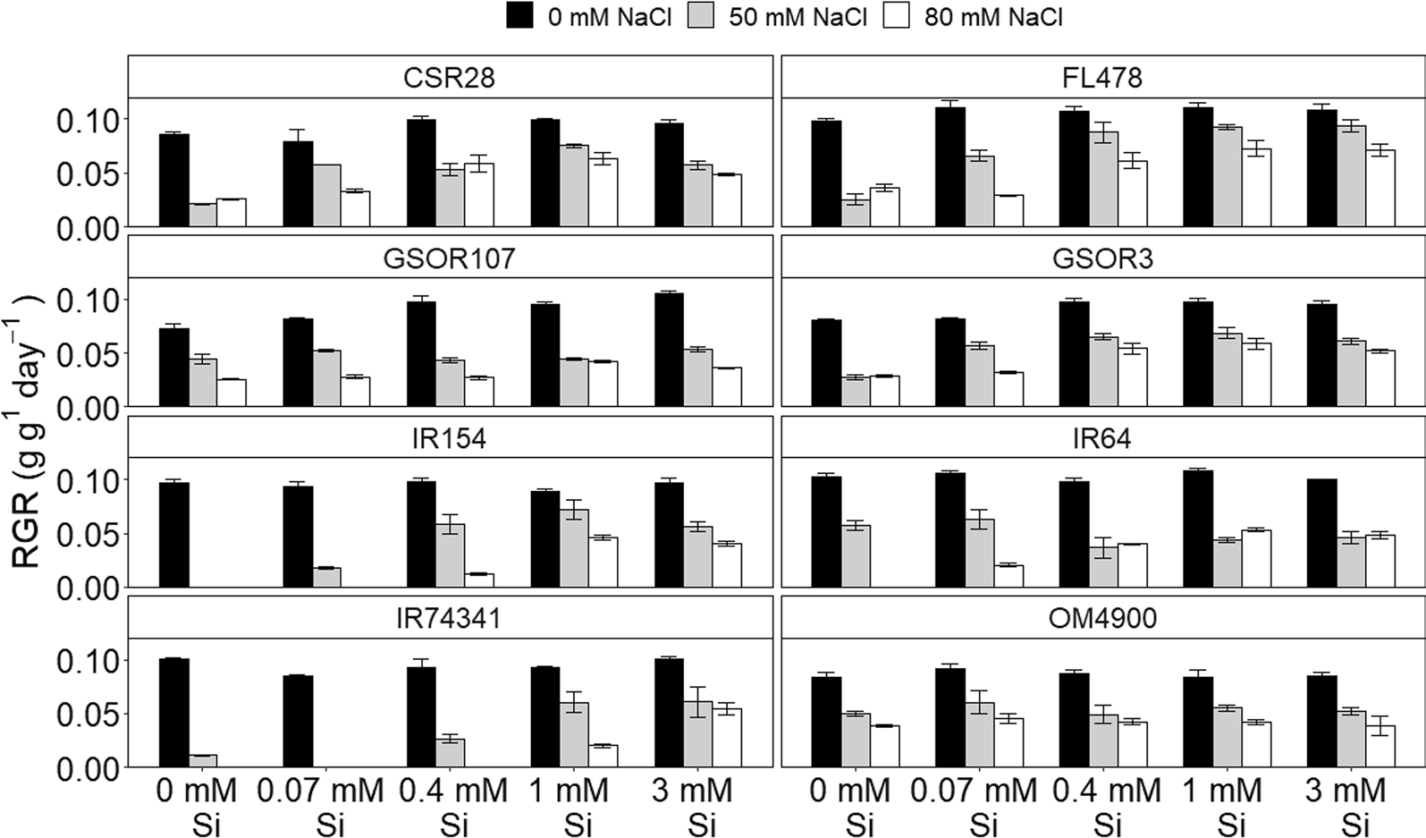
Growth response of 8 different rice cultivars to salinity and Si. Plants were grown in hydroponics for 30 d at 0, 0.07, 0.4, 1 and 3 mM added Si and with 0, 50 or 80 mM NaCl. Note the total absence of growth in salt sensitive varieties such as IR154 and IR74341 when the medium contains salt and low Si. Also evident is the lack of response to Si in varieties such as OM4900 and GSOR115. Data show means (N = 3) with standard deviations

The growth data in Fig. 3 show that the more salt-sensitive lines struggle to survive in low Si, saline conditions, but, to various degrees, they can be rescued by Si supplementation. In contrast, for other cultivars such as OM4900 there is no or little effect of Si (as was also seen in Fig. 1), irrespective of the salinity level, while an intermediate response is seen in GSOR3 where the beneficial effects of Si are primarily manifest at 80 but not at 50 mM NaCl. These more detailed data show that where plants do respond, the beneficial effect of Si for mitigating salt stress typically levels off when the external Si concentration reaches ~ 0.4 mM under 50 mM NaCl and when it lies between 0.4 and 1 mM for 80 mM NaCl (Fig. 3; Additional file 1: Table S3). However, this ‘critical’ value for maximum Si effect may be higher (~ 1 mM) in salt sensitive lines such as IR154 (at 80 mM) and IR74371 (at both 50 and 80 mM NaCl).

To assess how the external Si and NaCl conditions impact on tissue Si, root and shoot Si contents were analysed and values are shown in Table 2. Salinisation per se induced a large raise in tissue Si levels. This is particularly evident for shoot Si and occurred at all external Si concentrations. Although some papers report a salinity-induced reduction in tissue Si (e.g. Ahmed et al. 2019) many others show findings similar to ours, i.e. a substantial increase in Si (Mahdieh et al. 2015; Abdel-Haliem et al. 2017; Lekklar et al. 2019). As of yet, we lack a mechanistic explanation for it. It does argue against Si translocation being (mostly) transpiration-driven; transpiration is greatly reduced in response to both salinisation (Sultana et al. 1999; Moradi and Ismail 2007) and Si fertilisation (Gao et al. 2006; Farooq et al. 2015) yet shoot Si levels dramatically increase (Table 2; Additional file 1: Table S3). Alternatively, changes in Si transporter activity may underpin the increase in Si accumulation, with Abdel-Haliem et al. (2017) reporting that salt stress increased *Lsi1* and *Lsi2* expression in Si supplemented plants, although expression was decreased in salt-stressed plants without Si.

**Table 2:** Shoot and root Si levels averaged across 8 different rice varieties

Si (mM)	Na (mM)	Shoot Si (% DW)	Root Si (% DW)
0	0	0.38 ± 0.09	0.25 ± 0.05
	50	0.78 ± 0.15	0.25 ± 0.08
0.07	0	0.45 ± 0.05	0.22 ± 0.05
	50	0.82 ± 0.19	0.32 ± 0.06
0.4	0	1.03 ± 0.18	0.35 ± 0.10
	50	2.44 ± 0.47	0.56 ± 0.30
	80	2.72 ± 0.40	0.73 ± 0.39
1	0	1.70 ± 0.40	0.54 ± 0.11
	50	3.03 ± 0.86	0.73 ± 0.15
	80	3.98 ± 0.92	1.45 ± 1.03
3	0	2.55 ± 1.25	1.20 ± 0.71
	50	4.53 ± 0.85	2.34 ± 1.60
	80	4.97 ± 0.64	1.43 ± 0.61

Si was measured in plants grown for 30 days in media with 0, 0.07, 0.4, 1 or 3 mM Si and 0, 50 or 80 mM NaCl

Table 2 further shows that with 0.4 mM Si in the external medium, a level of Si where mitigation is maximum for most genotypes (see Fig. 3), the corresponding average level of root Si is 0.56% (value range of 0.47 to 0.76%; Additional file 1: Table S3). Thus, assuming the mitigating effect of Si is primarily root based, it is tempting to conclude that ~ 0.56% root Si suffices to maximise its benefits. However, maximum Si efficacy in salt-sensitive lines such as IR154 and IR743 requires around 1 mM external Si (Fig. 3), which corresponds to a root level of around 0.73% (value range of 0.56 to 0.88%). In other words, salt sensitive lines require ~ 30% more root Si for Si-induced mitigation of salt stress compared to more tolerant lines such as GSOR3, CSR28 and FL478.

In all, these findings suggest that (a) root Si is more important than shoot Si in protecting rice from salinity damage, (b) root Si levels of around 0.5 to 0.9% suffice to maximise the mitigating effects of Si and (c) salt sensitive lines require 30–40% more root Si than tolerant lines to achieve these benefits. Thus, while previous studies predominantly focussed on the role of shoot Si where salt stress is concerned (Abdel-Haliem et al. 2017; Farooq et al. 2019; Lekklar et al. 2019), or in other cases did not determine tissue Si levels (Gong et al. 2006; Shi et al. 2013; Flam-Shepherd et al. 2018), further studies on roots may help reveal the mechanistic basis for the mitigating effect of Si.

### The Impact of Si on Biomass and Yield in Soil Grown Plants

The above data give a useful foundation regarding the ambient (i.e. externally supplied) and internal tissue levels of Si that are necessary to achieve relief of salinity stress during moderate or severe salt stress. In an agronomic setting, it is important to determine the external levels of Si required to optimise growth improvement under salt stress, whilst insights into the ‘critical values’ will facilitate estimation of the amount of Si that needs to be replenished in order to prevent soil depletion of Si. To obtain estimates of these values in soil-like environments as opposed to hydroponic systems, and hence increase the practical relevance of our findings, a number of experiments were repeated using pot grown plants. Furthermore, plants were grown to maturity to allow us to quantify Si impact not only on biomass but also on grain yield.

Table 3 shows how biomass and yield were affected by Si and salinity after cultivation at 4 different salinities with electric conductivity (EC) of 0, 4, 6 and 8 dS m^−1^, and 3 different levels of 0, 90 and 130 kg ha^−1^ added Si. To normalise between growth experiments and cultivars, the data are expressed relative to the ‘no Si added’ control (absolute values for shoot and panicle biomass can be found in Additional file 1: Table S4). In general, salinity greatly suppressed plant vigour, and biomass changes in response to increased salinity largely reflected the data and findings obtained with our hydroponic system (c.f. Figure 3; Table 3). As in hydroponics, more growth reduction was recorded in pot grown sensitive lines such as IR154 and IR743 and less so in tolerant lines like CSR28 and IR64.

**Table 3:** Effect of Si fertilisation on rice biomass and grain yield

Cultivar	EC (dS m^−1^)	Shoot dry weight reduction (%)			Grain weight reduction (%)		
		Si (kg ha^−1^)			Si (kg ha^−1^)		
		0	90	130	0	90	130
IR64	4	24.6 ± 3.8	15.7 ± 4.4	15.1 ± 4.5	39.9 ± 2.8	21.9 ± 1.7	25.2 ± 3.9
IR64	6	33.8 ± 6.3	19.8 ± 5.4	18.4 ± 7.7	62.9 ± 16.7	42.8 ± 23.9	58.7 ± 18.2
IR64	8	43.2 ± 4.9	36.8 ± 13.9	19.1 ± 0.7	70.1 ± 18.7	75.5 ± 12.4	69.5 ± 12.2
CSR28	4	21.2 ± 6.6	12.3 ± 6.2	8.7 ± 7.4	12.5 ± 7.2	18.0 ± 12.8	21.5 ± 9.6
CSR28	6	26.1 ± 9.4	6.0 ± 9.0	9.7 ± 9.5	64.0 ± 9.8	52.0 ± 14.0	59.7 ± 6.9
CSR28	8	34.9 ± 19.0	10.9 ± 1.8	12.6 ± 14.5	71.9 ± 5.4	55.3 ± 9.3	65.3 ± 11.3
IR74371	4	34.5 ± 5.9	22.4 ± 5.1	16.5 ± 2.3	44.6 ± 6.3	42.9 ± 9.6	31.4 ± 9.2
IR74371	6	55.3 ± 16.2	42.2 ± 10.9	30.0 ± 4.0	62.6 ± 14.9	72.5 ± 2.6	75.0 ± 10.1
IR74371	8	69.0 ± 12.3	60.4 ± 15.9	48.7 ± 8.5	90.6 ± 3.9	91.2 ± 4.6	81.9 ± 9.2
IR83383	4	19.1 ± 8.5	15.3 ± 1.3	13.0 ± 10.3	24.3 ± 27.0	26.8 ± 17.5	28.0 ± 6.4
IR83383	6	38.7 ± 10.9	24.5 ± 10.0	22.2 ± 8.5	50.4 ± 9.1	44.5 ± 5.7	51.4 ± 8.8
IR83383	8	37.2 ± 6.2	40.4 ± 12.1	30.6 ± 7.9	63.2 ± 9.6	71.8 ± 15.9	73.8 ± 12.1
IR154	4	27.8 ± 16.4	29.7 ± 16.6	11.4 ± 7.7	45.8 ± 4.7	32.9 ± 20.9	28.7 ± 18.0
IR154	6	21.5 ± 1.8	40.0 ± 16.5	24.5 ± 9.0	77.0 ± 1.5	65.4 ± 16.1	39.0 ± 14.0
IR154	8	52.3 ± 11.5	50.3 ± 3.7	23.4 ± 8.9	82.2 ± 9.5	70.0 ± 12.6	57.7 ± 1.5
OM4900	4	20.6 ± 5.7	37.7 ± 4.3	45.0 ± 7.5	38.2 ± 17.6	50.2 ± 18.9	77.9 ± 3.0
OM4900	6	31.7 ± 6.9	50.7 ± 5.3	63.6 ± 16.0	73.1 ± 17.0	62.6 ± 8.5	92.2 ± 5.9
OM4900	8	32.5 ± 11.6	62.8 ± 7.6	74.6 ± 4.8	72.6 ± 7.3	84.5 ± 8.7	98.2 ± 2.1

Plants were soil grown on four levels of salinisation (EC of 0, 4, and 6 dS m^−1^) and three levels of Si fertilisation (0, 90 and 130 kg ha^−1^). The effect of Si is expressed as percentage reduction, relative to the ‘no Si added’ condition

One-way ANOVAs showed a significant positive impact of Si addition in limiting the salinity induced growth reduction in the case of three cultivars: IR64 (at EC = 6 and 8 dS m^−1^), IR743 (at EC = 4 dS m^−1^) and IR154 (at EC = 8 dS m^−1^). Though not significant at the 5% level, the CSR28 plants also showed a dose dependent and consistent trend toward growth rescue by Si. In contrast, the moderately salt tolerant OM4900 behaved differently: not only did it show a relatively large growth reduction under salt stress (as in hydroponics, Fig. 3), its growth was actually *reduced* significantly by Si supplementation at all three levels of salinity (EC = 4, 6 and 8 dS m^−1^), although there was no effect of Si addition for this variety in hydroponics (Fig. 3). Statistical tests on changes in grain yield showed significant mitigation of salt-induced yield reductions in IR64 (EC = 4 dS m^−1^), in CSR28 (EC = 4 dS m^−1^), and IR154 (EC = 6 and 8 dS m^−1^). No discernible influence of Si supplementation was found in the IR743 and IR833 cultivars, but as was seen for plant biomass, Si had a detrimental effect on OM4900 yield (EC = 4 and EC = 8 dS m^−1^).

Ahmed et al. (2019) found the beneficial effect of Si during salt stress in rice was similar for shoot dry weight and yield. We found that the impact of Si supplementation on mitigating salt stress was consistent across the two traits of biomass and yield for IR154, CSR28 and OM4900, but less so for IR64 (where yield rescue was only seen at EC = 4 dS m^−1^ while biomass rescue occurred at EC = 6 and 8 dS m^−1^), and not at all for IR74371, which showed biomass rescue but no effect of Si on yield. This suggests that it can be challenging to predict the beneficial effects of Si for rescuing yield under salt stress from measuring biomass alone; the most complete picture of the ability of Si to mitigate the impacts of salinity on the performance of a cultivar will come from measuring both biomass and yield.

### The Economic Feasibility of Si Supplementation

Our data show that Si can positively impact on both biomass and yield production in several cultivars (e.g. IR64, IR154, IR743 and CSR28). At the same time, Si does not appear to affect either growth or yield in other cultivars such as IR833, whereas it can even have a negative influence, as seen with the OM4900 variety. These different responses are clearly going to impact on the utility and efficacy of applying Si as a mitigation for salt stress. For example, the above data strongly suggest that in the case of OM4900 cultivation, Si supplementation is likely to be counter-productive and for cultivars like IR833, negative impacts are unlikely but the lack of measurable Si-induced growth promotion under salt stress would mean it was a waste of money.

For cultivars where Si did improve yield (IR64, IR74371 and IR154), Table 3 allows us to estimate yield improvement at the two Si supplementation levels. For these three cultivars, EC = 4 dS m^−1^ salinity caused on average a 43% drop in yield when no Si was added (background Si levels were equivalent to ~ 1 kg ha^−1^). This percentage reduced to ~ 30% and ~ 24% respectively when 90 or 130 kg ha^−1^ Si are applied. Thus, Si application at 130 kg ha^−1^ would generate a ~ 45% improvement relative to the no Si condition. In contrast, the average yield reductions for EC = 6 dS m^−1^ and EC = 8 dS m^−1^ would be around 65% and 80% in the absence of added Si. These values would change to around 60% and 55% for EC = 6 dS m^−1^ when Si is supplied at 90 or 130 kg ha^−1^ whereas the equivalent values at EC = 8 dS m^−1^ would be 80% and 70% for 90 and 130 kg ha^−1^ respectively. Overall, these numbers show that Si rescue is substantial at low level salt stress, but almost absent when it is moderate or severe.

Using field conditions that included a limited amount of water stress, Flores et al. (2021) suggested that foliar applications of intermediate levels of Si may be economically viable for rice. Likewise, a literature inventory by Alvarez and Datnoff (2001) concluded that Si fertilisation would likely be economically viable in most rice-producing countries. However, neither of these studies was based on specific, experimentally imposed, stress conditions and/or assessed the impact of different rice cultivars. To assess the applicability of Si as a commercially viable approach, a generalised costing model has been developed (Additional file 2: ‘Costing model’), based on a number of assumptions (see Suppl. data). Si fertiliser cost depends on its form; blast furnace slags have very low (2–5%) Si contents and can contain many other chemical components that can impact on plant growth. It is therefore not considered here. Likewise, rice straw is often used as a cheap form of Si fertiliser on many small holder farms, but this contains variable amounts of Si in addition to other chemical components and thus is not considered here. Na-, K- and Ca-silicates contain 20–25% Si and command prices of $500–1000 per tonne Si (e.g. https://www.alibaba.com/showroom/wollastonite-price.html). This equates to an extra production cost of $50–120 ha^−1^ when applying 90–130 kg ha^−1^ Si supplementation. The proportional impact of this cost varies according to production system.

In large (> 25 ha) farms in China or the USA, yields typically reach of 7–8 t ha^−1^ in non-stressed conditions. These farms do not use Si fertilisation, but the ambient Si availability is unknown. Assuming yields of 7 t ha^−1^, production value of around $3600, and costs of around $2200 ha^−1^ (Zhang et al. 2019), such farms would generate profit margins of about $1400 ha^−1^. In this scenario, a salinity induced yield reduction of 43% (see above) would lower sales income to $2050 (3600–1550), creating a loss of $150 ha^−1^. In the case of Si responsive cultivars, Si supplementation would restore production value to $2750 and consequently to profits of $495 ha^−1^. Fertiliser costs would reduce this to $430–450 ha^−1^. Moderate (EC = 6 dS m^−1^) and severe (EC = 8 ds m^−1^) stress would further eat into earnings generating a loss irrespective of the production system. It is important to point out that these calculations are made on the basis of yields rather than biomass; salt-induced biomass reductions are generally less severe than yield reductions (Table 3) and therefore apparent profitability would be achieved for EC6 and EC8 salinity levels in large farm production conditions.

However, much of the world’s rice production takes place in small holder farms, with 400 million people in Asia alone involved in growing rice on farms smaller than 2 ha (IRRI 2016). Such small holder farms typically have lower yields (2–3 t ha^−1^) and the cost–benefit analysis is very different. For example, Pathok and Deka (2019) estimate Indian average production costs per hectare (assuming 3 t ha^−1^ yield) of around $450 against a paddy sales price of ~ $625 ha^−1^ (based on the governmental minimum support price) generating a farmer’s income of ~ $175 ha^−1^. A salinity-induced yield reduction of 43% causes a net loss of around $30 ha^−1^, even in the presence of Si, and is clearly not sustainable. Using Fijian numbers of paddy sales price ($1930 ha^−1^) and production costs ($1700 ha^−1^; Bong 2017), results in a slightly higher farmers income of around $230 ha^−1^. But in this case too even mild salinity leads to an overall loss which, if a minimal extra cost of $50 is added for Si supply, amounts to $280 ha^−1^. In other countries, where low production, small holder-dominated rice cultivation prevails, very similar numbers populate cost–benefit analyses.

## Conclusions

The plant science literature has seen an explosion in the number of publications reporting the benefits of Si. This element appears to have positive properties that relate to all aspects of plant physiology, including abiotic stresses such as drought, heat, cold, flooding and metal toxicity, and biotic factors such as tolerance to pathogens and herbivory (see Singh et al. (2020), Thakral et al. (2021), and Thorne et al. (2020) for recent reviews). The mitigating effects of Si with respect to salt stress have been studied for decades, especially in rice (Matoh et al. 1986; Yeo et al. 1999; Gong et al. 2006). Most of these studies typically focused on the impact of Si on biomass in a specific cultivar (Gong et al. 2006; Farooq et al. 2019; Lekklar et al. 2019) whereas field studies frequently involve application of unrealistically high levels of Si supplementation (Mauad et al. 2016; Ullah et al. 2018; de Tombeur et al. 2021).

Work from this study shows that there is great variability in the benefits of Si addition, when ambient Si levels are low, for the alleviation of salinity stress, with rice varieties that are negatively impacted, those that do not respond, and others that show positive effects. Furthermore, the data suggest that Si efficacy is greater in more salt tolerant varieties. Thus, it is imperative that cultivar-specific data are collected in studies aiming to assess the benefits of Si supplementation in alleviating salt stress. Our results also suggest that changes in biomass are not necessarily good predictors of yield when determining the effects of Si fertilisation, so data on both parameters may be needed. In terms of practical applications, it would be very useful for such studies to include evaluations of the economic feasibility of Si supplementation, especially with reference to differing cultivation and production systems. The relatively simple cost–benefit model presented here is based on greenhouse studies and a small set of basic assumptions that can easily be adjusted for various economic parameters. Clearly, the actual financial gains and losses will be sensitive to multiple edaphic and climatological factors and will require data from specific cultivars, preferably in the form of field trials. In contrast, more general trends revealed by our modelling are less likely to depend on local conditions and include the notion that Si application is likely to be more profitable in high production systems and also at lower levels of salinisation.

## Methods

### Plant Growth Using Hydroponics

Rice seeds were germinated in sand. After 7 d, plants were transferred to standard Yoshida hydroponic medium and grown for another 10 d. Subsequently, plant weights were recorded and plants were exposed to hydroponic standard medium (control) or media that were supplemented with 50 or 80 mM NaCl to induce salinity stress. Various levels of Si (0, 0.07, 0.4, 1, or 3 mM) were applied by adding Na-silicate. Where appropriate, Na levels were normalised to 3 mM using NaCl. The hydroponic medium was renewed once per week and treatments lasted for 30 d, after which total plant, shoot and root fresh weights were determined. Plants were cultivated in a greenhouse with the following conditions: a 12 h photoperiod which consisted of natural daylight augmented with artificial light to 600–1000 µmol m^−2^ s^−1^. Day and night temperatures were 28 and 24 °C respectively. Relative growth rates (RGRs) were calculated as ln_t2_-ln_t1_/t_2_-t_1_ where t_1_ is the initial weight (g) and t_2_ the final weight. At least 3 biological repeats were carried out.

### Plant Growth in Soil

To simulate rice cultivation in soil, seeds were germinated in sand and after 7 d, six seedlings were transferred to a 10 L box which contained 7 kg of substrate of the following composition: 75% John Innes compost #2, 15% Coarse Vermiculite, 10% Sand (16:30 Silica Sand). Soil salinisation at 0, 4, 6 and 8 dS m^−1^ electric conductivity (EC) was achieved by adding 0, 400, 600 or 800 mL of a 50 mM NaCl solution in 8 instalments (twice a week) per box. Silicon fertilisation at 0, 90 and 130 kg ha^−1^ was achieved by adding 0, 720 or 1040 mg Si per box (800 cm^2^ surface) in the form of Na-silicate. XRF measurements (see below) showed low background Si content of around 0.1 mM (soil water basis), equivalent to around 1 kg ha^−1^. Silicon was applied in two doses, after one week and 5 weeks. Plants were grown in a greenhouse with 12 h day/night temperatures of 22 and 28 °C, ambient relative humidity and lighting with a minimal level of 500 µmoles m^−2^ s^−1^ for 6 months after which all shoot tissue was removed by cutting at the root:shoot junction for determination of plant and panicle weights. Three biological repeats were carried out.

### Flame Photometry Sample Preparation and Analysis

Shoots and roots of plants were separated and DW obtained after 48 h drying at 80 °C. Tissue was extracted for 48 h using 5 mL of CaCl_2_ (20 mM). Extract Na^+^ content was determined using a Sherwood 410 flame Photometer (Cambridge UK).

### Tissue Silicon Measurements

Silicon contents were was measured by portable X-ray fluorescence spectroscopy (P-XRF) using the method of Reidinger et al. (2012). Dried leaf and root material was ball-milled (Retsch MM400 Mixer mill, Haan, Germany) for 3 min at a frequency of 20 Hz. Ground material was pressed at 10 tons into pellets using a manual hydraulic press with a 13 mm die (Specac, Orpington, UK). Si analysis (% Si dry weight) was performed using a Nitron XL3t900 GOLDD XRF analyser (Thermo Scientific, Winchester, UK). For XRF calibration, silicon-spiked synthetic methyl cellulose (Sigma-Aldrich, product no. 274429) was used. To avoid signal loss by air absorption, the analyses were performed under a helium atmosphere (Reidinger et al. 2012).

### Statistical Analyses

All experiments consisted of at least 3 biological repeats and data are presented as means with standard deviation. To assess the effect of Si on biomass and yield of hydroponically and soil grown plants one-way ANOVAs were performed using p < 0.05.

## Supplementary Information

**Additional file 1. Table S1**: Germplasm used in this study. **Fig. S1**: correlations between relative growth rate (RGR) and tissue Si for plants grown hydroponically. **Table S2**: Tissue Na+ concentrations for plants grown hydroponically. **Table S3**: Tissue Si contents for plants grown hydroponically. **Table S4**: Shoot and panicle dry weights for plants grown in compost.

**Additional file 2. Costing model**: contains the data used to determine the economic feasibility of Si fertilisation.

## Data Availability

The datasets used and/or analysed during the current study are available from the corresponding author on reasonable request.
